# Serum C-reactive protein predicts early mortality in hospitalized patients with HBV-related decompensated cirrhosis

**DOI:** 10.1097/MD.0000000000005988

**Published:** 2017-01-27

**Authors:** ShaoMing Zhu, Yulituzi Waili, XiaoTing Qi, YueMei Chen, YuFeng Lou, Bo Chen

**Affiliations:** a Department of Ultrasonography, the First Affiliated Hospital, College of Medicine, Zhejiang University, Zhejiang; b Department of Clinical Laboratory, The Sixth Affiliated Hospital of Xinjiang Medical University, Xinjiang; c Department of Clinical Laboratory, The People's Hospital of Xinjiang Uygur Autonomous Region, Tianshan District, Urumqi; d Department of Clinical Laboratory; e Department of Hand Surgery, The First Affiliated Hospital, College of Medicine, Zhejiang University, Zhejiang, China.

**Keywords:** C-reactive protein (CRP), decompensated cirrhosis (DeCi), hepatitis B virus (HBV), model for end-stage liver disease score

## Abstract

The serum C-reactive protein (CRP) is an inflammatory marker. The aim of the present study was to elucidate whether CRP could serve as a potential surrogate marker for 30-day mortality in hospitalized patients with HBV-related decompensated cirrhosis (HBV-DeCi).

This was a retrospective cohort study that included 140 patients with HBV-DeCi. All patients were followed up for 1-month. A panel of clinical and biochemical variables were analyzed for potential associations with outcomes using multiple regression models.

The serum CRP was significantly higher in nonsurviving patients than in surviving patients. Multivariate analysis demonstrated that CRP levels (odds ratio: 1.047, *P* = 0.002) and the model for end-stage liver disease score (odds ratio: 1.370, *P* = 0.001) were independent predictors for mortality.

Serum CRP is a simple marker that may serve as an additional predictor of 1-month mortality in hospitalized patients with HBV-DeCi.

## 1 Introduction

Chronic hepatitis B virus (HBV) infection remains a major cause of liver cirrhosis in China, with a yearly incidence of decompensated cirrhosis of 3%.^[1]^ Decompensated cirrhosis is characterized by a number of complications, including ascites, hepatorenal syndrome, and upper gastrointestinal bleeding. Patients with decompensated cirrhosis have poor prognoses, and the 5-year survival of untreated patients with HBV-related decompensated cirrhosis (HBV-DeCi) is less than 15% compared with the ∼85% in patients with HBV-DeCi.^[2]^ Although the majority of patients with HBV-DeCi can be referred for liver transplantation, the shortage of donor livers and considerable cost make this approach unavailable for most patients at present.^[3,4]^ Therefore, discovery of a marker associated with the poor outcome of HBV-DeCi will help improve clinical management to mitigate the high rate of mortality.

The serum C-reactive protein (CRP) is an acute phase protein found in the blood stream the levels of which rise in response to inflammation and it has been extensively studied in coronary artery diseases, malignant tumors (including hepatocellular carcinoma), tissue necrosis, and bacterial translocation.^[5–8]^ Several studies have been performed on the association of CRP with the severity of inflammation in liver disease, such as fatty liver and chronic hepatitis C.^[9–12]^

Furthermore, 2 studies demonstrated that systemic inflammatory response was a major prognostic factor in patients with cirrhosis.^[13,14]^ In a previous prospective study, Cervoni et al^[15]^ suggested that CRP was able to predict 6-month mortality in patients with severe cirrhosis as defined by a Child–Pugh score B8 or higher. However, currently there are few markers that can predict 30-day mortality after hospital admission of patients with HBV-DeCi. In the present study, we hence investigated serum CRP as a predictor for 1-month mortality in a cohort of HBV-DeCi patients.

## 2 Materials and methods

### 2.1 Patients

The study was conducted as a retrospective follow-up of a cohort of 140 consecutive in-patients with HBV-DeCi from January 2014 to January 2015. Patients had to be HBsAg positive, previously diagnosed with HBV-related compensated cirrhosis, and presenting clinical manifestations of decompensated liver disease for the first time. There were no age- or gender-based exclusions. None of the patients had previous liver or other organ transplantation. DeCi was defined as in previous studies according to the presence of ascites, hepatic encephalopathy (HE), and/or variceal bleeding at the time of the study.^[16]^ Hepatorenal syndrome (HRS) and ascites were diagnosed using the criteria proposed by the International Ascites Club and American Association for the Study of Liver Disease, respectively.^[17,18]^ Patients with any of the following were excluded: acute hepatitis; hematologic disorders; malignancies such as hepatocellular carcinoma; pregnancy; concurrence of HCV, hepatitis D virus, human immunodeficiency virus infection, or autoimmune or other liver diseases. At baseline, for each patient, demographic and clinical data including age, sex, and complications related to liver disease such as ascites, HE, HRS, variceal bleeding, and clinical course in the hospital were obtained from the medical records and were recorded in a specified liver disease pro forma. Biochemical parameters including serum CRP, creatinine, total bilirubin, total protein, albumin, aspartate aminotransferase (AST), alanine aminotransferase (ALT), international normalized ratio (INR), hemoglobin, leukocyte counts, and platelet counts were recorded. All patients were followed-up for 1 month or longer to assess the 1-month in-hospital mortality. In addition, the model for end-stage liver disease (MELD) score and serological indexes (HBsAg, HBeAg, anti-HBc, and HBV DNA levels) were collected at baseline.

The study was performed according to the Declaration of Helsinki; the procedures were approved by the Ethics Committee of the First Affiliated Hospital of Zhejiang University College of Medicine. Written patient consent was not required.

### 2.2 MELD score

Liver disease severity was evaluated via the MELD score, which uses the patient's serum bilirubin and creatinine levels and the INR for prothrombin time to predict survival. The MELD score was calculated using the web site calculator (http://www.mayoclinic.org/gi-rst/mayomodel7.html).

### 2.3 Statistical analysis

All continuous variables were expressed as mean ± standard deviation (SD) or medians (range), and categorical data as percentages. Differences in variables were analyzed using Student's *t*-tests (for normally distributed data), or the Mann–Whitney *U* tests (for non-normally distributed data). Categorical data were evaluated by the χ^2^-test, as appropriate. Correlations between variables were examined using Spearman's correlation analysis. The diagnostic accuracy of prognostic variables was examined by receiver operating characteristic (ROC) analysis. Logistic regression analysis was employed to demonstrate the independent predictors for 4-week mortality rate of patients with HBV-DeCi. Statistical analyses were performed using the SPSS version 12.0 statistical package (SPSS Inc., Chicago, IL), and a *P* < 0.05 was considered statistically significant.

## 3 Results

### 3.1 Basic clinical and biochemical data

A total of 140 patients with HBV-DeCi were included in this retrospective study. Patient age ranged from 26 to 76 years (median: 53 years) and 108 (77.1%) of the patients were males. The baseline characteristics of these patients are summarized in Table 1. The serum CRP level**s** ranged from 0.8 to 146.8 mg/L (median: 9.25 mg/L). The CRP level was positively correlated with the leukocyte count (*r* = 0.360, *P* < 0.001). There were no correlations between the CRP level and the MELD score (*r* = 0.152, *P* = 0.072).

**Table 1:**
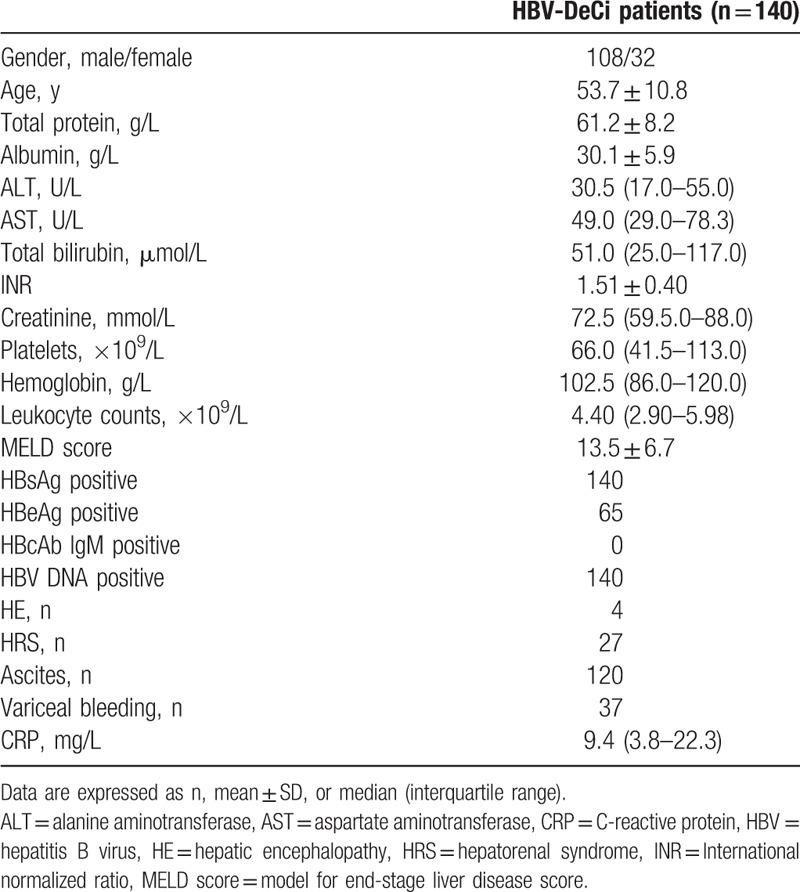
Baseline demographic and clinical characteristics of study participants.

### 3.2 Serum CRP levels were higher in nonsurviving patients than in surviving patients with HBV-DeCi

HBV-DeCi patients were divided into nonsurviving (n = 16) and surviving groups (n = 124). The clinical and laboratory characteristics of these patients are listed in Table 2. The nonsurviving patients had a higher MELD score, total bilirubin, creatinine, INR, leukocyte counts, and serum CRP levels compared with those in surviving patients. No significant differences in total protein, albumin, ALT, AST, gender, or age were detected.

**Table 2:**
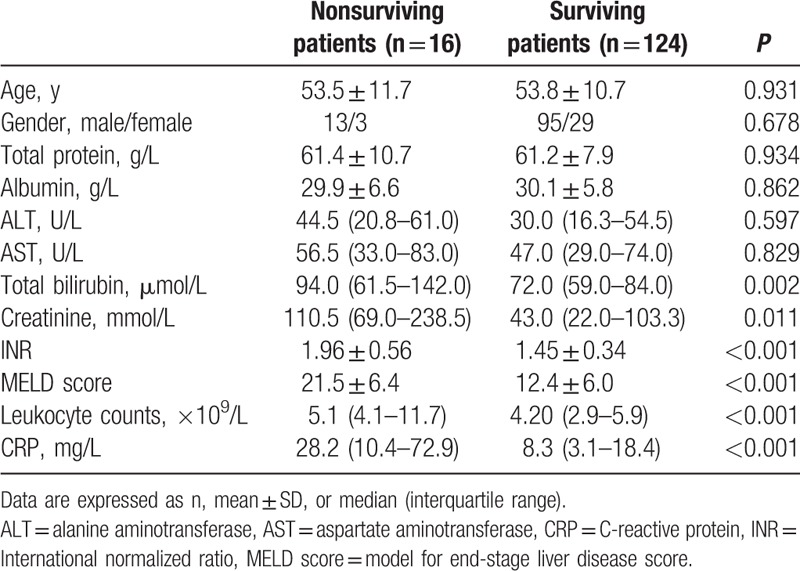
The clinical characteristics and differences in variables between nonsurviving and surviving patients with HBV-related decompensated cirrhosis.

### 3.3 Serum CRP as a predictor of 30-day mortality in HBV-DeCi patients

The patients were followed up for a median of 22 days (IQR: 12–77 days). During the follow-up, 16 patients died within 30 days due to upper gastrointestinal bleeding (n = 7), HE (n = 4), or HRS (n = 5). Univariate logistic regression analysis showed that high leukocyte count, high MELD score, and high serum CRP level were independent risk factors for 30-day mortality in HBV-DeCi patients. Multivariate logistic regression analysis identified both the MELD score and the CRP level as related to this mortality (Table 3). ROC curves were established to evaluate the relative efficiencies of the CRP level and MELD score for predicting the 30 days mortality (Fig. 1). The AUC was calculated as 0.850 ± 0.044 for the MELD score and 0.748 ± 0.066 for the CRP level (both *P* < 0.001). Baseline serum CRP levels were able to predict 1-month mortality with a similar performance to that of the MELD score (*P* = 0.309). When CRP and MELD were combined, the AUC was 0.916 ± 0.031.

**Table 3:**
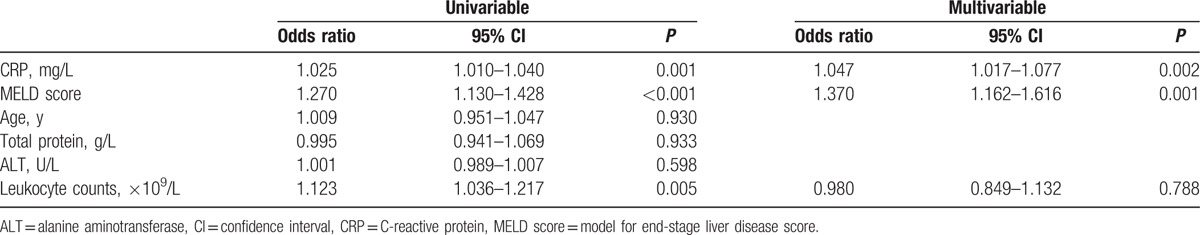
Risk factors associated with 1-mo mortality, as analyzed by Cox proportional hazards analysis.

**Figure:**
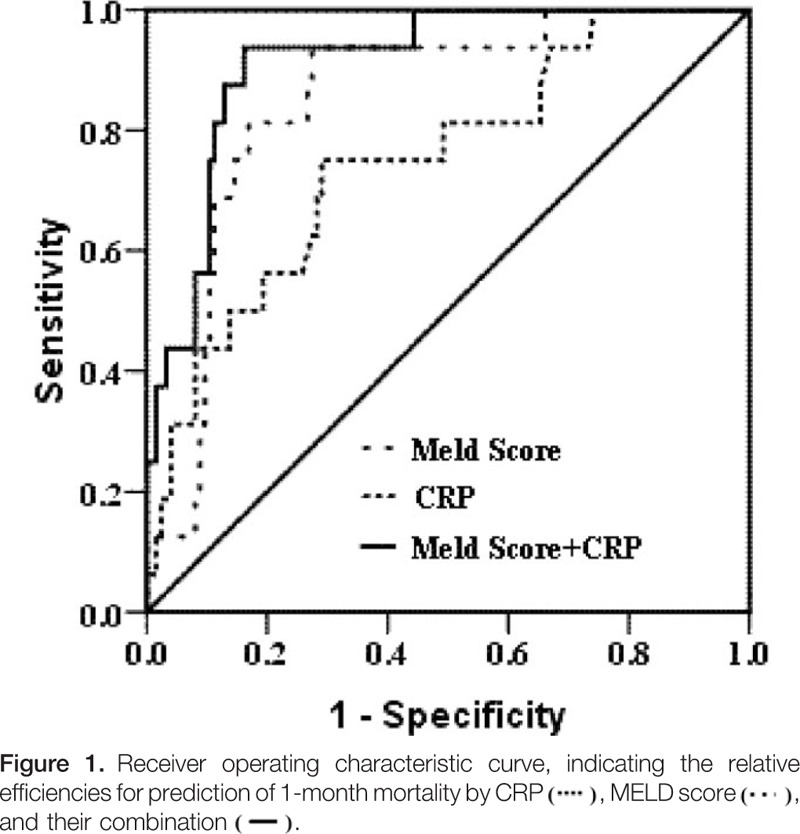


## 4 Discussion

The present study was conducted with the objective of establishing the role of the serum CRP as a prognostic indicator in patients with HBV-DeCi. We found that the CRP was significantly higher in nonsurviving patients than in surviving patients. More importantly, we also found that CRP was able to predict early mortality in HBV-DeCi patients.

The MELD score is known to signal risk for 3-month mortality and is used to assign priorities in the transplant waiting list for cadaveric livers.^[19]^ Our previous study reported that the MELD score was associated with prognosis of patients with acute-on-chronic liver failure.^[20]^ However, approximately 15% to 20% of candidates for liver transplantation are not well served by MELD. This is because important factors (i.e., HE, HRS, and upper gastrointestinal bleeding) that can affect the prognosis of patients were not taken into consideration in the MELD score.^[17,21–23]^ In the current study, we found no correlation between the MELD score and serum CRP, and this suggests that CRP may rescue some patients who are not prioritized by MELD (in patients with a low initial MELD score). Our result is identical to the data from Martino's group.^[24]^ Their study suggest that a model combining the MELD score and CRP variations may sort the candidates for liver transplantation better than the MELD score alone, but such a model warrants external validations. In our cohort, the predictive power of serum CRP was slightly lower than that of the MELD score, but the difference did not reach statistical significance. Moreover, the serum CRP involves only 1 marker, which makes it simpler and easier to achieve than the MELD score. A combination of CRP and MELD score could increase prediction efficiency to 92%.

The underlying mechanisms enabling the serum CRP to indicate possible outcomes in HBV-DeCi patients are not well established. Inflammation in patients with HBV-infected livers has been proven to be mediated by cytokines which may play a pivotal role in the pathogenesis of chronic HBV infection.^[25,26]^ Previous studies also have confirmed that systemic inflammation shown to favor serious complications such as variceal bleeding, encephalopathy, and acute-on-chronic liver failure.^[13,14,27]^A recent systematic review of 11,987 cirrhotic patients showed a 4-fold increase in the risk of mortality in the event of bacterial infection.^[28]^ In our cohort, 4 patients exhibited HE, 27 HRS, 37 gastrointestinal bleeding, and 120 ascites, which may have favored enteric bacterial infection. Furthermore, in this study, we found a strong positive correlation between serum CRP and the leukocyte count (*r* = 0.360, *P* < 0.001), which is a widely used marker of inflammation. CRP is synthesized in the acute phase of inflammation in response to interleukin-6, and an elevated CRP suggests the presence of hepatic inflammation as a response to liver injury. Hence, we assume that CRP is a marker of systemic inflammation, which strongly impacts on prognosis in DeCi patients.

A few limitations of this study warrant mention. First, our study was retrospectively conducted, which carried bias in selecting participants. Second, this was a single-center study, and sample size was relatively small. Our findings need to be verified in large multi-center and prospective studies. Finally, CRP was not kinetically monitored. We were not able to determine which CRP over time would perform better in predicting outcomes of HBV-DeCi patients.

## 5 Conclusions

In summary, our study demonstrates that CRP can function as an independent marker for predicting 1-month mortality in hospitalized patients with HBV-DeCi. Moreover, the serum CRP is an inexpensive, readily available, and reproducible test and has emerged as a marker of systemic inflammation. However, larger prospective trials are needed to validate these findings.
